# The History of a Case in Which Animals Were Found in Blood Drawn from the Veins

**Published:** 1834-01-01

**Authors:** 


**The History of a Case in which Animals were found in Blood drawn from the Veins of a Boy, with Remarks. By *J. Stevenson Bushnan*, F.L.S. Surgeon to the Dumfries Dispensary, &c. Octavo, pp. 74. Highley, 1833.**

This history occupies but two or three pages of the volume before us, the rest being dedicated to a very laborious research into ancient and modern writings, in quest of similar phenomena.

On the 4th of June, 1833, Mr. Bushnan was called to a poor boy residing on the banks of the Nith, who had been bled from the arm, and from whose blood issued fifteen worms, to the great consternation of the mother, and even of the neighbours. Mr. B. saw these worms, but, as the boy was labouring under the prevailing influenza, he drew six ounces of blood from the boy's arm, which he carefully covered with a basin, *and returned one hour afterwards*, when he discovered five animals swimming in the serum of the blood—all most vigorous and lively. On dividing the clot, it was curious to see the powerful efforts made by the animals to disengage themselves from it. From the crassamentum, he disengaged eight more, making fifteen obtained by himself—in all twenty-eight. Some of these he sent to his friend Mr. Rhind, of Edinburgh, an able helminthologist, who examined them, and reported on their nature. This report we shall here insert, as it is very concise.

"The animals I received from you lived with me two days in a little blood serum, when I had an opportunity of examining them most attentively. They are from about half an inch to six or eight lines in length; when dead the bodies relax and become about one inch long. They consist of an articulated body, of eleven joints, of a head with rudiments of four organs, (antennæ and palpi), with an appendage immediately below the articulation of the head, which is ciliated and very similar to the respiratory tubes at the other extremity. The tail terminates in two tubular bodies or stigmata having ciliated margins, these are the external respiratory organs; besides these, there are two or three bands on each side which are mere fleshy appendages. Within the articulated body, extending on each side from the tail to the head, the respiratory organs are distinctly visible with the aid of a microscope. These consist of a continuous tubular structure, of a pale silvery colour, through which the air passes. The colour of the animals is bright red.

These animals exactly correspond in structure and colour and size to the larvæ of the Tipula oleracea, which, in summer, is so abundantly found in ditch and river water. The eggs of these flies are very minute, and at a certain season of the year, are deposited in great numbers in running water, by the Tipula fly—well known by its long legs and slender body.

The worms cannot be mistaken for any of the Entozoa of the human body, or of other animals; because they have distinctly formed aerating organs, which intestinal worms never have been discovered to possess.

They are in many other points entirely different. Their red colour is a specific distinction, and, not likely to be accidental from the colour of the fluid in which they were found, and on which they appeared to feed. They also seemed to respire equally well in the blood serum as in water, for I could distinguish a constant succession of air globules in their respiratory tubes.

Believe me, very truly yours,

William Rhind."

Mr. B. adverts to the improbability of any deception in this case, from the animals having been in the cups previously to venesection, or introduced into the blood afterwards from design. We do not, ourselves, suspect any deception; but we certainly should have used more precaution on this occasion, and not allowed the blood to be out of our sight till properly examined. Mr. B. was anxious to procure some blood, by cupping, from the boy, but was unsuccessful. The boy resided on the banks of the Nith, the waters of which he was in the habit of drinking.

"He lingered long in a very hopeless condition, with every symptom of the greatest debility—a weak irritable pulse, seldom under one hundred and twenty in a minute, great prostration of strength, and severe wandering pains throughout the body, but especially in the back, œdematous swelling of the legs, a thickly-coated tongue, slight diarrhœa, sleepless nights, and not unfrequent delirium. He got better, however, and early in August was able to leave his room; and by the end of the month was pronounced recovered." 12.

Our author was, at first, led to believe that the foregoing case was unique; but there is nothing new under the sun. On wading through the records of the healing art, he found a multitude of analogous instances, no small number of which he has collected from various sources, and condensed into this little volume. To those whose investigations take a turn this way, the labours of Mr. Bushnan will be valuable; but to the great mass of our readers, the facts which we have here stated will be all they desire to know. Indeed we cannot help thinking that Mr. B. expended more labour and time in these researches than the subject deserved; since many of the records of worms in the blood are of dubious authenticity, and if true, of doubtful utility. By these remarks, we do not wish to detract from the zeal and labours of Mr. Bushnan, but only hope that they will next be directed to subjects of more general interest.

